# Extracorporeal Shockwave Therapy Increases Growth Factor Release from Equine Platelet-Rich Plasma *In Vitro*

**DOI:** 10.3389/fvets.2017.00205

**Published:** 2017-12-07

**Authors:** Kathryn A. Seabaugh, Merrilee Thoresen, Steeve Giguère

**Affiliations:** ^1^Department of Clinical Sciences, Colorado State University, Fort Collins, CO, United States; ^2^Department of Large Animal Medicine, University of Georgia, Athens, GA, United States; ^3^Department of Pathobiology & Population Medicine, College of Veterinary Medicine, Mississippi State University, Starkville, MS, United States

**Keywords:** extracorporeal shockwave therapy, platelet-rich plasma, equine, regenerative medicine, lameness, growth factors, tendon, ligament

## Abstract

**Introduction:**

Extracorporeal shockwave therapy (ESWT) and platelet-rich plasma (PRP) are common treatments for soft tissue injuries in horses. Shockwave triggers cell specific responses to promote healing. Growth factors released from PRP also promote healing. It has been hypothesized that greater growth factor release would amplify the healing process. The combination of ESWT and PRP could promote healing in injured tendons and ligaments in the horse. The objective of this study was to determine if application of shockwaves to PRP samples increases the concentration of transforming growth factor-β_1_ (TGF-β_1_) and platelet-derived growth factor ββ (PDGF-ββ) released from the platelets *in vitro*.

**Materials and methods:**

PRP was produced from blood drawn from six horses. The PRP from each horse was exposed to the following treatments: (1) positive control (freeze-thaw cycle), (2) untreated negative control, or shockwaves with either (3) a “standard probe” (ESWT-S) with a 2 cm focal width and medium energy density or (4) a “power probe” (ESWT-P) with a 1 cm focal width and high energy density. After each treatment, the samples were centrifuged, and the supernatant was harvested. The supernatant was then used for growth factor quantification *via* commercially available ELISA kits for TGF-β_1_ and PDGF-ββ.

**Results:**

Concentrations of TGF-β_1_ and PDGF-ββ in PRP that underwent a freeze-thaw cycle were significantly increased compared with all other treatments. Both ESWT-S and ESWT-P resulted in significantly increased TGF-β_1_ concentrations, 46 and 33%, respectively, when compared with the negative control. Both ESWT-S and ESWT-P resulted in significantly increased PDGF-ββ concentrations, 219 and 190%, respectively, when compared with the negative control.

**Discussion:**

These data indicate that the application of ESWT to PRP increases the expression of growth factors *in vitro*. This suggests that the combination therapy of local PRP injection followed by ESWT may stimulate release of growth factors from platelets after they have been injected into the area of injury.

**Conclusion:**

The combination of PRP and ESWT might result in synergism of two modalities previously utilized individually for tendon and ligament injuries in horses.

## Introduction

Extracorporeal shockwave therapy (ESWT) is a popular modality for the treatment of musculoskeletal injuries in the horse (1–6). Pulses emitted from the shockwave unit are transmitted into the tissues and stimulate healing of the targeted structure. It has been shown to improve the rate of healing of experimentally induced suspensory ligament desmitis in horses based on ultrasound assessment (2) and improve the prognosis of horses diagnosed with forelimb proximal suspensory desmitis returning to full work by 6 months (7). Although the mechanism of ESWT is not fully understood, it is believed to cause interstitial and extracellular responses leading to tissue regeneration and repair (8). Cell specific responses, including the release of growth factors such as vascular endothelial growth factor, insulin-like growth factor, transforming growth factor (TGF), bone morphogenic proteins, and other signaling molecules such as nitric oxide, have also been reported (9, 10).

Platelet-rich plasma (PRP) is also utilized for the treatment of tendon and ligament injuries in horses (11–20). PRP is a biomaterial which contains high concentrations of growth factors (21). Although traditionally an autologous blood product, allogeneic PRP products are becoming more popular in human medicine (22). This advancement has not yet become available in equine veterinary medicine and clinical use of equine PRP continues to utilize an autologous product. An attractive feature of PRP is that it can be deployed as a minimally manipulated stall-side treatment and produced with one of many commercial devices or kits available for the ambulatory equine practitioner. These commercial kits utilize filtration or centrifugation separation techniques to isolate and concentrate platelets and produce the PRP.

When the platelets are activated, growth factors are released from α-granules contained within the cells. These growth factors aid in healing and neovascularization of the injured tissues. Platelet activation may occur after PRP is delivered to injured tissues as a result of exposure to damaged collagen. This suggests that there is no need to activate PRP exogenously. Studies have in fact reported positive results following intralesional treatment with non-activated PRP (13, 14, 16, 20). By contrast, Textor et al. found only minimal activation of platelets when they were exposed to collagen *in vitro* (23). As a result, others have reported on the use of PRP activated exogenously (11, 12, 17, 19) using pretreatment activation techniques such as the addition of bovine thrombin or calcium chloride or exposure to a freeze-thaw cycle (24).

The evidence that supports the use of a platelet activation before injection raises the need to investigate other options available to manipulate PRP so to increase growth factor release. Utilizing a freeze-thaw cycle for activation negates the appeal of a stall-side treatment and activation with calcium chloride or bovine thrombin may result, respectively, in unnecessary laboratory steps and an unwanted inflammatory response to an exogenous protein (25). Clinically, the question has been raised whether shockwave therapy can be utilized to induce activation of PRP preparations to enhance growth factor release and possibly enhance the therapeutic efficacy.

Therefore, the objective of the study reported here was to quantify the concentration of platelet derived growth factor ββ (PDGF-ββ) and transforming growth factor β_1_ (TGF-β_1_) in PRP treated with ESWT. To accomplish this, we compared growth factor concentrations in the ESWT treated PRP samples to concentrations measured in freeze-thaw treated (positive control) and non-activated (negative control) PRP samples. We hypothesized that PRP preparations treated with ESWT would contain concentrations of growth factors comparable to those found in freeze-thaw activated PRP preparations.

## Materials and Methods

### Horses

The horses used for the study were all Quarter Horse mares aged 11–15 years (mean age = 13.5 years, median age = 14 years). The horses were all university owned and the institute’s animal care and use committee approved their participation in the study.

Platelet-rich plasma preparation—for the preparation of PRP, whole blood was collected from six adult horses. The site of venipuncture was clipped, prepared with 4% chlorhexidine, wiped with 70% isopropyl alcohol, and anesthetized with a 1 mL subcutaneous injection of 2% lidocaine. For each horse, blood was drawn into three 450 mL blood donor collection bags containing CPDA-1 anticoagulant (Teruflex Blood Bag System, Terumo Corporation, Tokyo, Japan). Blood bags were gently rocked during collection to ensure thorough mixing of blood with anticoagulant. Following collection, the lines were clamped with hemostatic forceps, and blood was transported to the laboratory in coolers containing ice packs. In addition, a 7 mL sample was collected into a blood tube containing EDTA for a baseline complete blood count performed on a blood analyzer (Heska Blood Analyzer, Loveland, CO, USA).

Working under a laminar flow hood and using sterile technique, blood from each horse was transferred to 50 mL tubes. Sequential centrifugation was used to concentrate the platelets from each horse. Tubes of blood were centrifuged for 10 min at 700 *g* and 4°C with no brake. The plasma was collected and placed into new 50 mL conical tubes and then centrifuged again for 5 min at 1,250 *g* at 4°C. The plasma supernatant was collected and centrifuged a third time for 5 min at 2,370 *g* at 4°C. The pelleted platelets from the second centrifugation were resuspended *via* repeated aspiration with a pipette and combined into one 50 mL tube. Plasma supernatant from the third centrifugation was considered platelet poor plasma, and a 50 mL aliquot was set aside while the resulting pelleted platelets were resuspended and combined with the platelets that were collected following the second centrifugation. A 15 μL sample of the platelet concentrate was collected and diluted with an equal volume of phosphate buffered saline and a platelet count was obtained using a blood analyzer (Heska Blood Analyzer, Loveland, CO, USA). The platelet count was used to determine the proper dilution volume, and platelet poor plasma was then added back to the sample to obtain a target platelet concentration of 1.0 × 10^6^ platelets/μL. The resulting platelet concentrate was well mixed, and 2.5 mL aliquots were placed in multiple 3 mL glass tubes immediately following preparation.

### Sample Treatments

**Figure 1:**
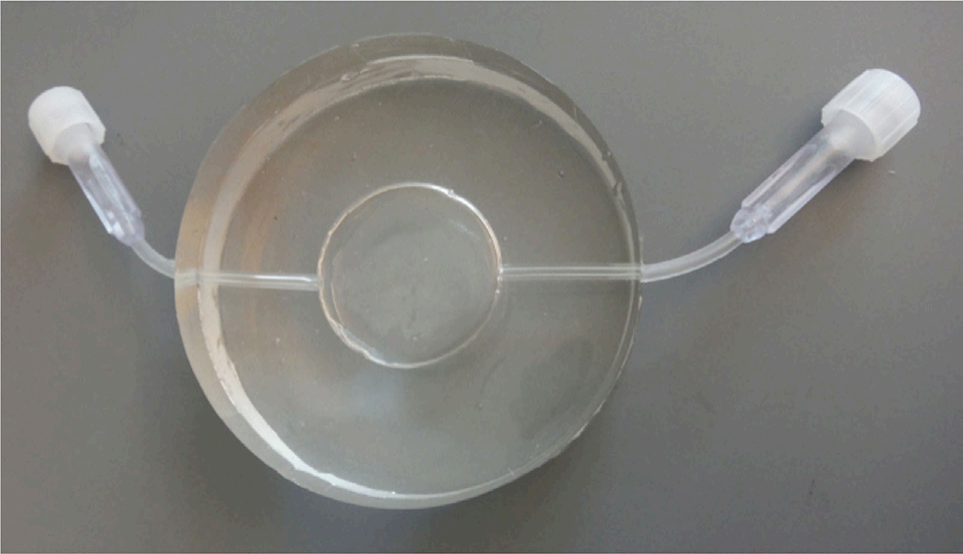
Silicone gel pad measuring 60 mm × 22 mm with a 25 mm × 2 mm reservoir and two ports to allow injection and withdrawal of platelet-rich plasma. The wall thickness on either side of the reservoir measures 10 mm.

**Figure 2:**
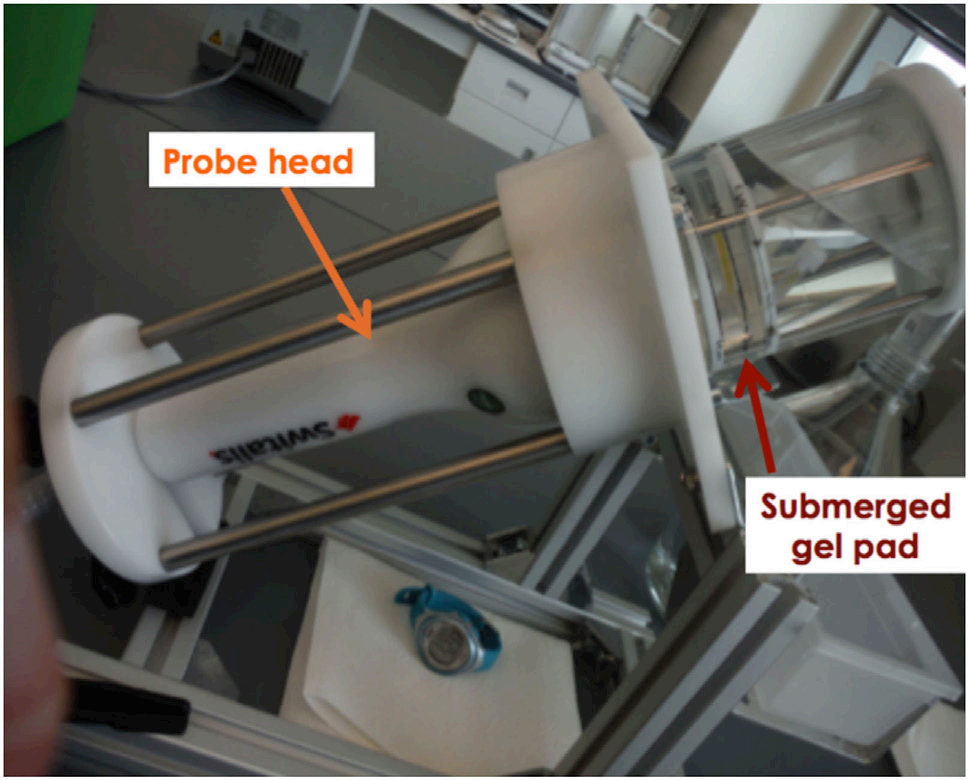
The probe of the shockwave unit was fixed into the treatment device. A watertight container was affixed to the probe head. The gel pad was submerged into the water bath for all treatments. Shocks were only administered for the shockwave treatment groups.

The individual aliquots of PRP from each horse were subjected to one of four treatment conditions as follows: (1) positive control (single freeze/thaw cycle), (2) negative control (resting), (3) ESWT-S (standard probe), or (4) ESWT-P (power probe). Individual aliquots were aspirated from the glass tubes with an 18 gauge, 9 cm (3.5 in.) needle and 3 mL syringe. The sample was then injected into a 2.5 mL hollow space in the center of a ballistic gel pad measuring 60 mm × 22 mm (Figure 1). The gel pad was designed to mimic the acoustic impedance of soft tissue. The pad was submerged into a water bath attached to the ESWT probe (Figure 2) so that shockwave parameters would mimic those established by the manufacturer. The treated samples were exposed to shockwaves from an electrohydraulic shockwave machine (Neo Shockwave Device, Nucleus Regenerative Therapies, Kennesaw, GA, USA) while submerged in the water bath. Both the ESWT-S and ESWT-P samples were submerged in the water bath, and 300 pulses at 23 kV and 2 Hz were administered. The standard probe (ESWT-S) administered 0.12 mJ/mm^2^ (energy density) with a wide focus (20 mm diameter) delivering 8.5 mJ total energy per pulse. The power probe (ESWT-P) administered 0.28 mJ/mm^2^ (energy density) with a narrow focus (10 mm diameter) delivering 4.4 mJ total energy per pulse (Table 1). Energy and pulse frequencies were selected based on data collected during a pilot study. Both the positive and negative control samples remained submerged for 2 min and were then withdrawn from the water bath. They were not exposed to any shockwaves. The distance between the probe head to the PRP sample in the gel pad was adjusted in all cases to simulate a treatment depth of 25 mm.

**Table 1:** Characterization of each probe used for treatments.

Probe	Focus length	Width	Energy density (mJ/mm^2^)	Total energy (mJ/pulse)
Standard probe [extracorporeal shockwave therapy (ESWT)-S]	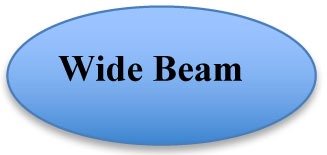	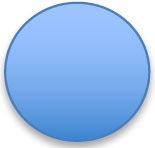 20mm	0.12	8.5
Power probe (ESWT-P)	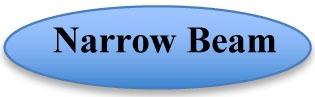	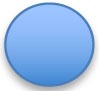 10mm	0.28	4.4

After each treatment, the sample was withdrawn from the reservoir within the gel pad and placed into 1.5 mL polypropylene microcentrifuge tubes. All samples sat at room temperature for 120 min after treatment to accommodate any delay in release of growth factors following treatment (26). After 120 min, the positive control samples were frozen at −20°C for 12 h. The treated samples and negative controls were centrifuged (21,000 *g* for 3 min), and the supernatant was harvested. After freezing, the positive controls thawed at room temperature and were then centrifuged as described earlier, and the supernatant was harvested. The supernatant from all samples was stored at −80°C and subsequently thawed and analyzed for growth factor concentrations.

### Growth Factor Analysis

Quantification of growth factors concentrations was performed on the supernatant using commercially available ELISA kits for PDGF-ββ (Quantikine human PDGF-ββ ELISA, R&D Systems Inc., Minneapolis, MN, USA) and TGF-β_1_ (Quantikine human TGF-β_1_ ELISA, R&D Systems Inc., Minneapolis, MN, USA). The use of these kits has been previously described in horses (27–31), and the manufacturer’s protocols were followed for each assay. Based on manufacturer’s protocols, the samples for the TGF-β_1_ ELISA were diluted fourfold to ensure that all samples were within the range of the standard curve of the assay. The resulting values were multiplied four times to account for the dilution.

### Statistical Analysis

Normality of the data and equality of variances were assessed using Shapiro–Wilk’s test and Levene’s test, respectively. The data were not normally distributed. Therefore, the effect of treatment (ESWT-S, ESWT-P, and positive and negative controls) was assessed using the Friedman repeated measures analysis of variance on ranks. Multiple pairwise comparisons were done using the Student–Newman–Keuls method. Statistical significance was set at *P* < 0.05.

## Results

Baseline complete blood counts were within normal limits for five of the six horses. One horse had a platelet count below normal (80 × 10^3^/μL, reference range 100–600 × 10^3^/μL), but there were no clinical signs consistent with thrombocytopenia. Starting PRP concentrations as well as final PRP concentrations, white blood cell count and red blood cell counts are represented in Table 2.

**Table 2:** Initial platelet-rich plasma (PRP) concentration is the concentration of platelets after sequential centrifugation.

Horse	Starting PRP concentration	Final PRP information		
	PLT (×10^3^/μL)	WBC (×10^3^/μL)	RBC (×10^6^/μL)	PLT (×10^3^/μL)
1	2,214	0.6	0.06	900
2	1,730	0	0.04	954
3	1,712	0.4	0.08	1,194
4	2,888	0.8	0.06	1,210
5	1,194	1.4	0.06	952
6	3,120	1	0.04	1,014
Mean	2,143.00	0.84	0.06	1,037.33
Median	1,972.00	0.80	0.06	984.00

*Final PRP concentration is after platelet poor plasma had been added back to achieve a concentration as close to 1.0 × 10^6^/μL. White blood cell count and red blood cell counts are also reported for the final PRP sample*.

Concentrations of TGF-β_1_ in ESWT-S and ESWT-P treatment groups (median = 2,101 and 2,000 pg/mL, respectively) were significantly higher than the negative controls (median = 1,485 pg/mL); however, TGF-β_1_ concentrations from the positive controls (median = 7,063 pg/mL) were significantly higher than all other treatments. There was no statistically significant difference in TGF-β_1_ concentrations between the two ESWT treatment groups. The median and quartile concentrations for TGF-β_1_ are listed in Table 3.

**Table 3:** Median (25th and 75th percentiles) concentration of transforming growth factor-β_1_ (TGF-β_1_) released into the supernatant following treatment.

Group	Concentration (pg/mL)	Increase relative to negative control (%)
Positive	7,063 (5,040–10,042)	355 (291–445)
Extracorporeal shockwave therapy (ESWT)-S	2,101 (1,488–3,357)	46 (32–56)
ESWT-P	2,000 (1,669–2,785)	33 (20–59)
Negative	1,485 (1,012–2,501)	

*Relative increases of growth factor concentrations compared with negative (resting) controls are also listed*.

Concentrations of PDGF-ββ in both ESWT treatment groups (median = 566 and 405 pg/mL, respectively) were significantly higher than the negative controls (median = 174 pg/mL); however, PDGF-ββ concentrations from the positive controls (median = 4,107 pg/mL) were significantly higher than all other treatments. There was no significant difference in PDGF-ββ concentrations between the two ESWT treatment groups. The median and quartile concentration for PDGF-ββ are listed in Table 4.

**Table 4:** Median (25th and 75th percentiles) concentration of platelet-derived growth factor-ββ (PDGF-ββ) released into the supernatant following treatment.

Group	Concentration (pg/mL)	Increase relative to negative control (%)
Positive	4,107 (3,718–4,229)	1,295 (1,051–4,017)
Extracorporeal shockwave therapy (ESWT)-S	566 (408–724)	219 (86–418)
ESWT-P	405 (331–593)	190 (57–340)
Negative	174 (94–307)	

*Relative increases of growth factor concentrations compared with negative (resting) controls are also listed*.

## Discussion

This study demonstrates that the application of ESWT to PRP preparations enhances the release of growth factors from platelets *in vitro*. Based on these results, ESWT may offer a valuable practical alternative to enhance PRP growth factor release following injection into the site of injury. This is of significant benefit when PRP is produced stall side so that diagnosis and complimentary treatment modalities can be administered during a single visit.

To mimic the process of field treatment of an equine soft tissue injury as closely as possible, we designed our shockwave experiments so that the sound field would not be influenced by reflection, diffraction, or secondary shockwaves (32). Therefore, the base material of the gel pad, silicone, was selected due to its density, which is similar to human tissue (33). In addition, the use of a water bath immersion allowed us to maintain sound field parameters equal to those performed and reported by the manufacturer (32). This chamber technology also allowed for the simple retrieval of the sample of PRP for posttreatment analysis. The authors propose this model as a standard for future studies involving biologic materials in attempt to closely mimic *in vivo* tissue reactions.

When activated, platelets release multiple growth factors and cytokines from their α granules (29, 34) including TGF-β_1_ and PDGF-ββ, which have been repeatedly evaluated in many other related studies (23–25, 35, 36). The authors chose to evaluate concentrations of TGF-β_1_ and PDGF-ββ because these growth factors are found in the high concentrations in equine platelets (24) giving us the opportunity to more accurately quantify concentration changes relative to our treatment groups. Furthermore, release kinetics of TGF-β_1_ and PDGF-ββ from platelets have been most extensively studied and have been shown to play critical roles in the regeneration and healing of damaged tissues (37, 38).

Multiple methods of exogenous activation of equine platelets have been evaluated (24). The addition of calcium chloride or bovine thrombin can result in platelet activation as can exposure to a freeze-thaw cycle. Calcium chloride results in a significantly greater release of PDGF than bovine thrombin or a freeze-thaw cycle but all methods resulted in equal release of TGF (24). In this study, the authors chose to employ a single freeze/thaw cycle as the benchmark for maximum growth factor release from PRP. The negative control group represented minimal platelet activation and growth factor release concentrations. The positive and negative control groups provided a spectrum of platelet activation to which our shockwave groups could be compared. The study demonstrated that the freeze/thaw cycle resulted in significantly higher growth factor concentrations within the supernatant than the concentrations obtain following the shockwave treatments. This could be interpreted as a freeze/thaw cycle may be a better therapeutic option when utilizing PRP clinically. However, as mentioned earlier, freezing and thawing the PRP sample would preclude its use as a stall-side therapy. It has also been suggested that a freeze/thaw cycle results in platelet lysing and complete degranulation (36, 39) rather than platelet activation. It has been shown that platelets may continue to release growth factors for several days after reaching the injury site, because the half-life of platelets has been estimated to be 5–7 days (40). Complete degranulation of platelets may be undesirable.

Clinically it is unknown whether exogenous activation is superior as no randomized controlled studies have compared activated and inactivated PRP. In this study, treating PRP samples with ESWT resulted in an increase in growth factor concentration *in vitro* suggesting that shockwaves may have activated platelets and aided in the release of PDGF-ββ and TGF-β_1_ in the medium. It should also be recognized that since ESWT is, in itself, a therapy for soft tissue injuries, combination with PRP not only may increase growth factor release but it will also provide direct therapeutic effects.

This study provides useful information for equine veterinarians but does have limitations. Are we comfortable extrapolating these data from *in vitro* to *in vivo*? Clinically, when PRP is injected into a soft tissue structure it is typically injected into a “space” (i.e., a core lesion of a tendon or ligament) with ultrasound guidance. We can visualize the deposition of the PRP with ultrasound. We feel that immediately after injection the PRP remains as a focus of fluid similar to the volume of PRP contained within our experimental chamber. Therefore, although it should be done cautiously, these *in vitro* data are encouraging regarding the *in vivo* effects of ESWT on PRP. The second limitation is that we do not know the exact mechanism by which EWST activates the platelets. ESWT research would suggest that the sudden increase and decrease in pressure would apply mechanical stimulation to the platelet thereby activating it. It is not believed that the shockwaves rupture the platelet. One reason for this assumption is that *in vitro* mesenchymal stem cells treated with ESWT did not have reduced viability (41) and only had minimal changes to morphology (42). Second, if the platelets were ruptured by the shockwave pulses, we would have expected a greater release of growth factors as was found in platelet lysate products (36).

The results of this study support the use of ESWT in combination with PRP for treatment of tendon and ligament injuries in horses. This *in vitro* study suggests that the combination therapy of PRP injection followed by ESWT may stimulate release of growth factors from platelets after they have been injected into the area of injury. Thus, the combination therapy might result in synergism of two modalities previously utilized individually for tendon and ligament injuries in horses.

## Author Contributions

KS was the primary investigator in this research. She organized and executed the research and was primarily responsible for manuscript creation. MT assisted with data collection and manuscript review. SG assisted with statistics and manuscript review.

## Conflict of Interest Statement

The authors have no conflicts of interest to declare. Funding, shockwave equipment, and silicon gel pads were provided by the shockwave manufacturer Nucleus Regenerative Therapies. Research, conclusions and manuscript production was performed independently from any influence from the sponsor.
